# Interdisciplinary sport injury research and the integration of qualitative and quantitative data

**DOI:** 10.1186/s12874-023-01929-1

**Published:** 2023-05-03

**Authors:** S.E Hausken-Sutter, K Boije af Gennäs, A Schubring, S Grau, J Jungmalm, N Barker-Ruchti

**Affiliations:** 1 Department of Food and Nutrition, and Sport Science, University of Gothenburg, Box 300, 405 30 Gothenburg, Sweden; 2 Department of Sport Science, Malmö University, Malmö, Sweden; 3 Institute of Sociology and Gender Studies, German Sport University Cologne, Cologne, Germany; 4 School of Health Sciences, Örebro University, Örebro, Sweden

**Keywords:** Complexity, Football, Youth, Qualitative, Quantitative, Methodology

## Abstract

**Background:**

To understand and prevent sport injuries, scholars have employed different scientific approaches and research methods. Traditionally, this research has been monodisciplinary, relying on one subdiscipline of sport science and applying qualitative or quantitative research methods. Recently, scholars have argued that traditional approaches fail to address contextual components of sport and the nonlinear interactions between different aspects in and around the athlete, and, as a way forward, called for alternative approaches to sport injury research. Discussion of alternative approaches are today taking place, however, practical examples that demonstrate what such approaches entails are rare. Therefore, the purpose of this paper is to draw on an interdisciplinary research approach to (1) outline an interdisciplinary case analysis procedure (ICAP); and (2) provide an example for future interdisciplinary sport injury research.

**Methods:**

We adopt an established definition and application of interdisciplinary research to develop and pilot the ICAP for interdisciplinary sport injury teams aiming to integrate qualitative and quantitative sport injury data. The development and piloting of ICAP was possible by drawing on work conducted in the interdisciplinary research project “Injury-free children and adolescents: Towards better practice in Swedish football” (the FIT project).

**Results:**

The ICAP guides interdisciplinary sport injury teams through three stages: 1. Create a more comprehensive understanding of sport injury aetiology by drawing on existing knowledge from multiple scientific perspectives; 2. Collate analysed qualitative and quantitative sport injury data into a multilevel data catalogue; and 3. Engage in an integrated discussion of the collated data in the interdisciplinary research team.

**Conclusion:**

The ICAP is a practical example of how an interdisciplinary team of sport injury scholars can approach the complex problem of sport injury aetiology and work to integrate qualitative and quantitative data through three stages. The ICAP is a step towards overcoming the obstacles of integrating qualitative and quantitative methods and data that scholars have identified.

**Supplementary Information:**

The online version contains supplementary material available at 10.1186/s12874-023-01929-1.

## Introduction

Traditionally, sport injury researchers study injury aetiology in youth athletes from the perspective of one scientific discipline (e.g., exercise physiology, biomechanics; sport psychology; sport sociology). Broadly speaking, researchers of these disciplines follow distinctive assumptions of what an injury is and what research questions, ethical stances, research methods and interpretations and explanations of results are most appropriate to researching injury aetiology in youth athletes [1–3]. Biomedical scholars, for instance, often regard an injury to be related to identifiable individual physical factors and apply quantitative methods to test if components such as muscle strength previous injury, and growth and maturation are related to injury aetiology [4–6]. Sport sociologists often understand sport injury as a socially constructed phenomenon and apply qualitative methods to interview youth athletes about the coach-athlete relationship and/or observe contextual aspects such as the training environment [7, 8]. To study aspects such as injury experiences, consequences and perceptions, sport psychology researchers oftentimes use either quantitative [9], qualitative [10] or mix qualitative and quantitative methods [11].

The predominant monodisciplinary approach to sport injury research has provided extensive knowledge on injury aetiology in youth athletes. In recent years, however, several sport injury scholars have critiqued the traditional monodisciplinary approach and suggested a turn to *complexity approaches* to account for the multifaceted nature of sport injury aetiology [12–17]. The key argument is that contemporary research has not been accounting for the nonlinear interactions between different components across different dimensions, such as interactions between people and the physical and social environments, and thus, does not consider the unpredictable, fluid, and flux nature of sport injuries. Instead, the scholars suggest a framework away from risk factors towards identifying risk patterns and looking deeper into the complex nature of sport injury aetiology [12, 13, 15–17]. To achieve this goal, however, scholars consider *how* to best address complexity differently, and best practice examples are a work in progress for sport injury research. To address this gap and to contribute to the current discussion on alternative approaches we propose that *interdisciplinarity* offers potential. We have adapted and applied the definition of interdisciplinarity based on Julie Klein and William H. Newell [18], who have significantly influenced the field of interdisciplinary research in the past 40 years. These scholars define interdisciplinarity as a research process that addresses a complex phenomenon that cannot be dealt with adequately by a single scientific discipline. To fit Klein and Newell’s definition to the team context within which we conducted interdisciplinary research, we adapted Klein and Newell’s [18] definition which in this article involves collaboration of researchers specialising in different scientific disciplines and methodological approaches, and the application of both qualitative and quantitative methods.

The need for interdisciplinarity in sport injury research was first called for by Burwitz et al. [19] in the 1990s. Since then, several sport science scholars have argued that research on athlete health and wellbeing requires a holistic and multidimensional approach, where scholars from different disciplines collaborate [20–22]. The rationale is that different scientific perspectives and research methods have established important insight into sport injury aetiology and can thus address a greater range of components that influence sport injury aetiology. The different disciplinary insights offer a means to facilitate an integrated understanding and discussion of sport injuries in relation to individual players’ context and situation, which has the potential to extend existing insights [19, 22, 23]. For example, and as demonstrated by sport science scholars Schofield, Thorpe, and Sims [24], their bringing of qualitative sociological data into dialogue with quantitative physiological data helped the team to draw novel conclusions as to which athletes were struggling with health problems, which eventually led to new insight and a return to the empirical data for a second stage of analysis. Such new and integrated insight into athlete health is necessary to develop prevention strategies that are more effective in addressing the components of sport injury aetiology. To that end, this paper contributes with a piloted procedure on how to work in an interdisciplinary team with qualitative and quantitative data in sport injury research. Specifically, the purpose of this paper is to draw on an interdisciplinary research approach to (1). outline an interdisciplinary case analysis procedure (ICAP); and (2). provide an example for future interdisciplinary sport injury research.

### Interdisciplinary research and implications for data analysis

Interdisciplinary scholars Klein and Newell [18] define interdisciplinary research as:

- a process of answering a question, solving a problem, or addressing a topic that is too broad or complex to be dealt with adequately by a single discipline or profession … [interdisciplinary research] draws on disciplinary perspectives and integrates their insights through construction of a more comprehensive perspective [18 p3].

Interdisciplinarity thus constitutes both a research approach and a process that is developed for the study of complex systems [23]. A key aspect of interdisciplinary research is integration: “…crafting an integrated *synthesis* of the separate parts that provide a larger, more holistic understanding of the question, problem or issue at hand” [18 p12; emphasis in original]. Detailing this definition, interdisciplinarians Repko, Szostak and Buchberger [25] outline that integration is a cognitive process, where the researcher(s) evaluate disciplinary knowledge from multiple scientific perspectives and create a more comprehensive understanding of the problem under study based on the disciplinary knowledge. The common ground is, according to several interdisciplinary scholars, necessary for integration of disciplinary insight to be possible [25, 26]. For interdisciplinary sport injury research, we took this to mean that a team of disciplinarians, could collaborate, share, and integrate disciplinary knowledge, and engage in a discussion during which qualitative and quantitative data could be integrated.

The interdisciplinary research approach outlined above may seem familiar to scholars conducting mixed methods research in, for example, health research and sport psychology [27, 28]. Mixed methods research does indeed often aim to integrate qualitative and quantitative methods and data to gain broad and deep understanding and to generate unique insight into multifaceted phenomena [27, 29, 30]. However, the type of interdisciplinarity proposed in this paper differs from the mixed methods research approach by involving strategies for dealing with an array of ontological, epistemological, and contextual challenges that often exist or emerge when a team of disciplinarians collaborate. For example, interdisciplinary teams in sport science research can experience, and have experienced problematic power relationships, language barriers, and misunderstandings that complicate the integration of qualitative and quantitative data if these issues are not dealt with in the team [22, 24, 31]. Such teamwork and related onto-epistemological differences have received sparse attention in mixed methods research [32–34]. Therefore, to account for these differences, interdisciplinarity does not only involve strategies for integrating methods and data, but also for integrating disciplinary knowledge to create a more comprehensive understanding of the problem under study, which is necessary for integration to be successful [26].

With the potential and challenges of interdisciplinary research in mind, how can qualitative and quantitative data be integrated in an interdisciplinary research team context? As we could not locate established procedures for interdisciplinarity in sport science and sport injury research, we draw on suggestions of an applied interdisciplinary process developed by Newell and colleagues [26, 35], which constitutes integrative steps to guide researchers through the decisions made in the interdisciplinary process. According to these scholars, integration cannot follow an algorithm; rather, integration requires analytical reasoning and creative thinking as the interdisciplinary research process and its steps are iterative and complex [26, 35]. Moreover, being humble, respectful of, and acknowledging each other’s perspectives has been recognised as valuable cognitive skills when aiming to integrate knowledge and data across disciplinary borders [22, 36]. To successfully conduct integrated research, then, efforts beyond those associated with conducting high-quality disciplinary research and mixed methods approaches are necessary [26]. First, researchers need to understand a problem from different perspectives and disciplines. Second, researchers need to consider different disciplinary views and the methodological toolkits that the disciplines constitute. Finally, it is important that researchers embrace a holistic approach – an understanding of how disciplinary ideas and information relate to a problem and to each other. In sum, as the holistic thinking involved in interdisciplinary research opposes the traditional reductionist disciplinary strategy, interdisciplinary research is not “business as usual” [26 p262].

To develop an interdisciplinary case analysis procedure, which became the ICAP, we draw on research conducted in the interdisciplinary research project “Injury-free children and adolescents: Towards better practice in Swedish football (the FIT project) [37]. The purpose of the FIT project was to provide evidence-based interdisciplinary injury prevention strategies. The project aimed to produce a comprehensive and integrated picture of injury aetiology in a sample of male and female Swedish football players aged 10 to 19. The research team consisted of scholars from four scientific disciplines—biomechanics, sport medicine, sport sociology, and sport coaching. Based on the four scholars’ respective scientific expertise, qualitative data was generated through interview and observation-studies and quantitative data through biomedical measurements (kinematics/movement; strength; joint range of motion/flexibility; Peak Height Velocity (PHV)) and a longitudinal questionnaire study implementing an adapted version of the OSTRC-H questionnaire [38]. Upon completion of the studies, qualitative and quantitative data were analysed according to their respective disciplinary data analysis methods and quality standards (e.g., thematic analysis for qualitative interview and observation-data; statistical procedures for biomedical data). The next step was to perform integrated data analysis, which led us to the development of the ICAP.

### The Interdisciplinary Case Analysis Procedure (ICAP)

**Fig. 1:**
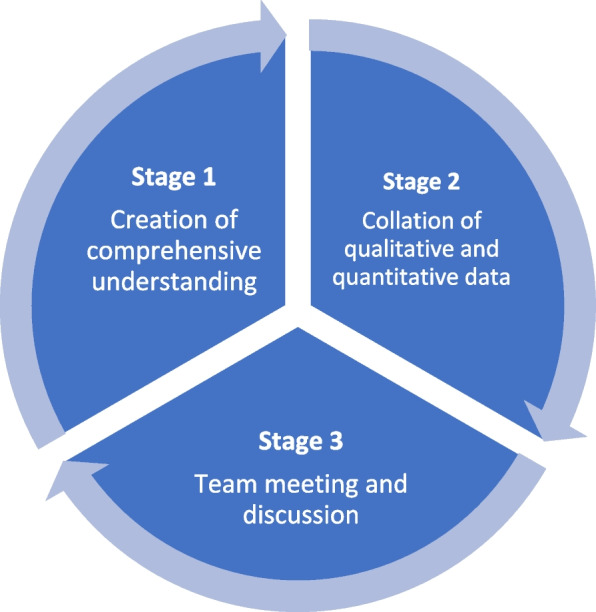
The three stages of the Interdisciplinary Case Analysis Procedure (ICAP)

The ICAP is a flexible, circular, and iterative procedure entailing three stages (see Fig. 1). The stages reflect the research process of a team of disciplinary researchers aiming to integrate data through an interdisciplinary data analysis procedure. In stage 1 and taking seriously the need for integration of disciplinary insights early in the research process, the aim is to create a comprehensive understanding of the phenomenon/a that the project team aims to study based on the scientific disciplines included in a project. In stage 2, qualitative and quantitative data, analysed according to their respective disciplinary standards, are brought together. Finally, in stage 3, the collated data is discussed through a team meeting consisting of the researchers representing the data included in step 2.

### Stage 1: Creation of comprehensive understanding

In stage 1, the aim is to create a comprehensive understanding of the problem that the project team intends to study based on the scientific disciplines included in the project [23]. To create comprehensive understanding, it is necessary that the team members find a common language, recognise conflicts and their unique strengths, and the disciplinary knowledge each member brings to the study [23]. In this stage, the either/or disciplinary thinking is replaced by both/and thinking requiring the disciplinarians to “think outside of the box” [26 p260].

For the FIT project to create comprehensive understanding of sport injury, we held several team meetings to discuss and reflect upon our different research approaches and understandings of sport injury aetiology. The meetings were carefully planned and led by the project leader, who aimed to be inclusive in type of language and making room for all disciplinary perspectives. We also reviewed diverse disciplinary literature relevant to sport injury, with a particular focus on youth football, to critically reflect upon onto-epistemological differences in sport injury research for the narrative review article we published together [39]. Through reviewing literature, we also considered the basic assumptions of complexity thinking, especially in relation to nonlinear interactions between different components in the athlete’s context. Moreover, the project was presented within and outside of academia to gain additional knowledge on disciplinary research approaches and sport injury aetiology in youth athletes. The planning and implementation of the FIT project’s four sub-studies also taught us more about the differences in qualitative and quantitative methods in relation to concepts such as recruitment, validity, and reliability. Finally, all researchers had the opportunity to participate in the respective studies, where, for example, the sport coaching researchers participated in the biomedical testing.

### Stage 2: Collation of qualitative and quantitative data

The aim of Stage 2 is to bring together qualitative and quantitative data in preparation for stage 3’s integrated discussion of injury aetiology.

For the FIT project, we focused on one single case of a female player aged 14 that had participated in all four studies included in the FIT project. This entailed two steps: First, individual analysis of the different datasets using suitable data analysis methods (i.e., thematic analysis for qualitative interview and observation-data; statistical procedures for biomedical data). Second, collation of the analysed data per research participant in a multilevel data catalogue in the form of an Excel document (see supplemental online file). The idea of this catalogue is to visualise and collate in a common “space” qualitative and quantitative data to provide a foundation for the integrated discussion in stage 3. The multilevel data catalogue entails six levels of information (see Table 1 for a simplified overview; for a more comprehensive description of the six levels, see the supplementary file).

**Table 1:** Stage 2 of the Interdisciplinary Case Analysis Procedure (ICAP). The table includes a simplified overview of the collation of quantitative and qualitative data for one 14-year-old female injured football player

Level 1	Perspective	General				Biomedical		Sociological	
Level 2	**Theme**	**Baseline information**			**Injury**^**a**^** information**	**Kinematics/ movement**	**Strength**	**Interview with player & coach**	**Observation of training sessions**
Level 3	**Type of data (examples)**	Age	Gender	Training	Diagnosis, duration, severity	Hip adduction range of motion (°)	Hip abduction / adduction ratio	Knowledge about injury and injury prevention	Communication between player and coach
Level 4	**Individual raw data (value or quote)**	14	Girl	7,5 h of football training and 6 h of handball training per week	Partial rupture left quadriceps, 3 weeks decreased or out of training	4,54	0,83	Athlete quote: *Injuries happen because of many different reasons. Mainly due to tough play *Coach quote: *Injuries happen because they [players] might be untrained, or train too much*	The training context is relaxed; there is little interaction between the coach and the players
Level 5	**Group reference or code**	14,7 ± 2,8	Girls			5,54 ± 2,10	0,86 ± 0,15	Injury related to tough play and overloading	Relaxed, laissez-faire coaching
Level 6	**Interpretation & evaluation**	Completed growth spurt, very high training volume (> 10 h); both football and handball			Overuse injury	Individual values are within 1 standard deviation (SD) of the mean value; e.g. no values outside of ± 1SD (Reference group: females aged 14–19)		The complexity of sport injury development is not mentioned, coach and athlete mainly focus on training aspects	Lack of specific feedback and steering to achieve desired outcomes (i.e., injury prevention, effective training, desired learning)

^a^Injury was defined based on a consensus statement from Fuller et al. [40 p193] where an injury is defined as: “Any physical complaint sustained by a player that results from a football match or football training, irrespective of the need for medical attention or time loss from football activities. An injury that results in a player receiving medical attention is referred to as a ‘‘medical attention’’ injury, and an injury that results in a player 13 being unable to take a full part in future football training or match play as a ‘‘time loss’’ injury”. Moreover, an overuse injury was defined as “caused by repeated microtrauma without a single, identifiable event responsible for the injury” [40 p194]

In level 1, to demonstrate the FIT project’s disciplinary perspectives, the multilevel data catalogue is divided into one biomedical (biomechanics, sport medicine) and one sociological (sociology, sport coaching) section. The purpose of level 2 is to show the different types of measurement and research methods employed under each disciplinary perspective. The columns in level 2 are divided into different biomedical- and sociology-themes (e.g., strength measurements; observation, interview). Level 3 specifies the type of data measured and generated for each of the themes. For example, for the strength theme, the hip abduction/adduction ratio is listed in separate columns. For the interview theme, topics such as “knowledge about injury and injury prevention” are listed. Level 4 contains data excerpts to demonstrate the type of qualitative and quantitative data from the individual analyses of the injured football player. Quantitative data is represented in numeric form (for example results from the strength measurements) while qualitative data is represented in textual form (for example quotes from the interview). Level 5 shows the reference value for qualitative and quantitative data. For the former, codes were given through a qualitative thematic analysis procedure [41]. For the latter, individual biomedical data was calculated and compared to the mean values of one reference group “females aged 14–19”. Finally, level 6 contains interpretation and evaluation of the qualitative and quantitative data in relation to reference values and literature. This level lays the most important groundwork for the team discussion and continuation of data integration for stage 3.

### Stage 3: Team meeting and discussion

In stage 3, the aim is for the researchers from the different disciplines included in the interdisciplinary project to meet and discuss the collated qualitative and quantitative data. According to Newell [26 p261], the goal of this interdisciplinary stage is to “achieve a balance among disciplinary influences on the more comprehensive understanding”, i.e., no disciplinary perspective should dominate the discussion. The qualitative and quantitative data about the complex problem (i.e., sport injury) is in this stage examined to “identify patterns of behaviour” [26 p261], or relationships (interactions) between different components in the system that influence injury aetiology.

For the FIT project, stage 3 was conducted through a team meeting consisting of researchers representing the scientific disciplines included in the project. The discussion was moderated by one of the researchers in the team, who had experience from the FIT project’s four sub-studies and knowledge of interdisciplinary research. The data catalogue containing analysed data served as the basis for the two-step discussion: First, each researcher presented interpretations of the analysis of data relevant to their disciplinary expertise. Their interpretations were related to the FIT project’s overarching aim and were not yet specific to a specific case/research participant. During each researcher’s statement of the analysed data, the other team members were invited to ask questions, which is argued to enable a deeper understanding of the problem at hand [42]. Second the different perspectives and data were related to the 14-year-old female player’s injury in a joint discussion. The integrated discussion was also a way to identify different patterns in the empirical data.

### Implications for interdisciplinary injury data analysis

As part of the process of developing and piloting the ICAP, we have encountered four issues that have implications for the use of the procedure and future research.

First, to facilitate the collation of qualitative and quantitative sport injury data in interdisciplinary research, we experienced that the different assumptions regarding disciplinary perspectives and qualitative and quantitative data require consideration early in the interdisciplinary research process. We propose that this consideration is vital as underlying ontological, epistemological, and methodological assumptions can complicate interdisciplinary research and integration due to misunderstandings and difficulties in reflecting and verbalizing these assumptions among members of interdisciplinary research teams. Therefore, the ICAP was, and needs to be part of a purpose-driven interdisciplinary research process that focuses on integration of disciplinary perspectives and research methods already in the planning and designing-phase of a project.

Second, as differences in assumptions influence how researchers define and research a phenomenon, it is necessary to facilitate collation through three circular, iterative, and pragmatic stages that enable teamwork across disciplinary borders. Indeed, working interdisciplinarily requires spaces, or “a community of research practice” [3 p56] within and through which the team can explore, negotiate, and reflect upon their commonalities and differences in scientific perspectives [43]. We have therefore found that it was of great importance that the team followed a procedure through which we met on a regular basis and had a team leader that supported methodological flexibility throughout the process. Such regular team meetings have indeed been found to facilitate the development of strategies that can help bring qualitative and quantitative materials together [24]. Following such a procedure does not, however, mean that working interdisciplinary is a strict and linear process. On the contrary, we did, for example, experience that we had to go back to stage 1 and learn more about concepts such as reliability, validity, credibility, generalisability, and transferability in relation to qualitative and quantitative methods [44] when interpreting the data in stage 3.

Third, and to further facilitate collation of qualitative and quantitative data in an interdisciplinary research team context, we noticed that the team benefitted from including a researcher with knowledge of interdisciplinary research and the different disciplines included in the project. We found this particularly important in stage 3 of the ICAP, when the team discussed the compiled data in relation to the injured player. When the discussion reached a dead-end, or when the disciplinarians misunderstood each other or the data, the interdisciplinary researcher moderator could clear up misunderstandings by, for example, pointing out how the different disciplines understand and interpret concepts differently and helping the team to find a common language. It occurs, for instance that qualitative and quantitative data contradict, which can be seen as a problem and an obstacle for integration [31]. Including an interdisciplinarian in the integration phase can, however, help the team use the contradictions in data to create new insight into the problem under study [31], which is key in Newell’s interdisciplinary process [26]. The idea of the *interdisciplinarian*, [26] or *interlocutor*, [30] as someone in the middle, who takes part in dialogue and conversation with the disciplinarians, can help the team see beyond their disciplinary borders, create unity, and refocus the team’s efforts towards constructive engagement in knowledge production [43]. Although the interdisciplinarian might not be able to eliminate possible power inequalities between the disciplinarians, paying attention to these boundaries and engaging the team in conversation can facilitate a common and interdisciplinary understanding of sport injury aetiology. For the FIT project, the interdisciplinarian helped the team to establish several aspects that needed further development, such as a need for a larger quantitative data set to be able to finalise the quantitative analysis as well as a need for additional cases to find patterns between cases. The team also realised the need for discussing the qualitative data in relation to findings and interpretations from similar qualitative research.

Fourth, we have noticed that successful integration requires a common understanding of what integration means in the team and where in the research process integration should take place. For the FIT project, integration involved a comprehensive understanding of sport injury aetiology in stage 1 [39], the collation of qualitative and quantitative data in one common space in stage 2 (the multilevel data catalogue), and an integrated discussion in stage 3 which together facilitated our interdisciplinary understanding of sport injury aetiology. There are, however, differences in degrees of integration [45]. Sometimes, for example, integration of knowledge and the collaborative process includes actors outside of academia and can lead to the creation of a new framework, which can generate a fundamental epistemological shift [36, 43]. Being clear in the beginning of a project on what, when, and how to integrate is key for sucessful collaboration across disciplinary boarders.

Finally, some methodological limitations need to be considered before conducting an integrated analysis procedure such as the ICAP. First, the ICAP is a complex procedure to carry out and requires more time, resources, and expertise than traditional analysis procedures. Second, there is a lack of research on the integration of qualitative and quantitative data in the interdisciplinary research context, and more research is needed on the integrated potential of such an approach and process. Finally, in the interest of better understanding the complexity of sport injury aetiology, there is a need to explore the pragmatic negotiations that an interdisciplinary research team needs to make when integrating seemingly opposing worldviews, methods, and data.

## Conclusion

The purpose of this paper was to draw on an interdisciplinary research approach to (1) outline an interdisciplinary case analysis procedure (ICAP); and (2) provide an example for future interdisciplinary sport injury research. The Interdisciplinary Case Analysis Procedure (ICAP) consists of a three-stage process that allowed us to create a more comprehensive understanding of sport injury aetiology, collate qualitative and quantitative data in a multilevel data catalogue and engage in an integrated discussion to identify patterns in the empirical data. Working interdisciplinarity is not business as usual and requires researchers to adopt certain cognitive skills that might be outside of their disciplinary comfort-zone. Creativity, flexibility, and openness are key such skills.

While we have developed the ICAP specifically for an interdisciplinary youth sport injury research project, the procedure is generic and can be applied in interdisciplinary research addressing other complex phenomena. For researchers who aim to adopt (and adapt) the ICAP, it is important to keep in mind that the procedure is not “just” about mixing or integrating qualitative and quantitative data, it includes strategies to integrate disciplinary knowledge and consider onto-epistemological differences throughout the whole research process. In so doing, the ICAP is a step towards overcoming the obstacles of integrating qualitative and quantitative methods and data that scholars have identified. It is our hope that sport science and other researchers will consider and apply ICAP in the interest of better understanding the complexities of a phenomenon under study.

## Supplementary Information

**Additional file 1.**

## Data Availability

The datasets used and analysed during the current study are available from the corresponding author on reasonable request.
